# Short-term olfactory deprivation reorganizes brain activity

**DOI:** 10.1192/j.eurpsy.2024.1293

**Published:** 2024-08-27

**Authors:** S. Tukaiev, I. Zyma, M. Makarchuk

**Affiliations:** ^1^Faculty of Communication, Culture, and Society, Institute of Public Health, Università della Svizzera italiana, Lugano, Switzerland; ^2^Institute of Biology and Medicine, Taras Shevchenko National University of Kyiv, Kyiv, Ukraine

## Abstract

**Introduction:**

Olfactory loss (including short-term) initiates neural reorganization processes in the brain, but the central mechanisms of this largely remain unknown.

**Objectives:**

We aim to conduct a neurophysiological study of neocortical mechanisms for the effects of olfactory sensation on functional activity of human brain under temporary obstruction of nasal breathing.

**Methods:**

123 healthy volunteers (76 female and 47 male students aged 18 to 23 years)participated in this study.EEG was registered during the rest state (5 min), olfactory blockage (5 min), under odor stimulation with the lemon essential oil (5 min) and renewal of nasal breathing (5 min). We estimated the spectral power density and the levels of coherence of all the frequencies from 0.2 to 35 Hz.

**Results:**

The onset of orthonasal olfactory sensory blockage was accompanied by an increase in the power of processes of local synchronization of beta frequency in the caudal regions of the brain, with simultaneous enhancement of the coherence of the theta band in parietal-occipital zones and a certain enhancement of the interfrontal and intrahemispherical left-side and right-side interactions in the beta2-subband. Thus, the sudden cessation of olfactory detection led to activation of thalamo-cortical loops and top-down control systems (search for the olfactory signal): that is an active orienting process (triangle of increase in F4-T4-P4 connections) with emotional coloring (the caudal localization of the coherence changes in the theta band). The prolongation of the nasal blockage, despite the possibility of activation of the retronasal route of odor perception and odorized air, was accompanied by a definite inhibition of distant interactions in the posterior regions of the brain in the theta-band and a significant decrease in right-brain long-distant and parietooccipital beta2-subband functional connectivity. Restoration of nasal breathing and olfactory perception is accompanied by sufficiently powerful activation of interhemispheric long and short distance information interactions in the theta1,2 and beta1 frequency bands.

**Conclusions:**

Our data indicated that cessation and restoration of olfactory perception lead to an increase cognitive activity, the development of memory processes, the current sensory and cognitive-emotional control of behavioral reactions, focusing attention, assessment of the significant stimulus.

**Disclosure of Interest:**

None Declared

